# Dr. Caroline Osborn Koenig

**Published:** 1917-03

**Authors:** 


**Dr. Caroline Osborn Koenig,** Buffalo 1904, of Buffalo, died at the Hahnemann Hospital of Rochester, Jan. 28, of pneumonia, after a brief illness. She was the classmate and wife of Dr. Edward C. Koenig, the Roentgenologist, the former home of both having been Tonawanda, where the funeral was held.

